# Supplementary material for: Patterns of Intron Gain and Loss in Fungi
Source: PLoS Biol. 2004 Nov 30;2(12):e422. doi: 10.1371/journal.pbio.0020422 (PMC532390; doi:10.1371/journal.pbio.0020422)
Supplement: Table S1 — Also available at http://genes.mit.edu/NielsenEtAl/. (4.3 MB ZIP). [file pbio.0020422.st001.zip › NielsenEtAl/html/272.html]

AN2963.1.NCU01323.1.MG06894.1.FG01910.1


```
 CLUSTAL W (1.82) Multiple Sequence Alignments - Introns Inserted


Sequence 1: NCU01323.1	1241 aa
Sequence 2: FG01910.1	1263 aa
Sequence 3: MG06894.1	1259 aa
Sequence 4: AN2963.1	1261 aa
Alignment Length: 1283 aa
Number Identitical Residues: 511 aa
Alignment Score (without introns) 27325


MG06894.1 	MGKLLRLELFN1FKSYKGHHTLLFGDSYFTSIIGPNGSGKSNS2MDAISFVLGIKSSHLR
NCU01323.1	MGKLIRLELFN1FKSYKGHHTLLFGDSYFTSIIGPNGSGKSNS2MDAISFVLGIKSSHLR
FG01910.1 	MGKLIRLELFN1FKSYKGHHVLLFGDSYFTSIIGPNGSGKSNS2MDAISFVLGIKSSHLR
AN2963.1  	MGKLIRLELYN1FKSYKGHHTLLFGDAYFTSIIGPNGSGKSNS2MDAISFVLGIKSSHLR
          	****:****:* ********.*****:**************** ****************

MG06894.1 	STHLKDLVYRGRVMKTSKINEDGSAAAPDTNGHTNGA-QNGDD~ESQPAT-RTDPKTAWV
NCU01323.1	STHLRDLVYRG-------------------------------H1PSQRSS-RNDPKTAWV
FG01910.1 	SAHLKDLVYRGRVLKTAKINDDGSAQTNGDA-----------N~GDEKAS-RGDPKTAWV
AN2963.1  	STNLRDLIYRGRVLRTSKVDADGNAVDRETEGVEPTQNEYDVE~PSQDASGTNDPRTAWV
          	*::*:**:***    ::. . ...:      .     .. . .  .: ::.  **:****

MG06894.1 	MAVYEDEAGDEQRWKRSITSQGASEYRINERVVTSQAYNEALEAENILIKARNFLVFQGD
NCU01323.1	MAVYEDDAGDEQRWKRTITNSGSSEYRINDRVVTAQQYNDALEAENILIKARNFLVFQGD
FG01910.1 	MAVYEDDAGEEQKWKRSITNQGASEYRINDRVVTAQQYNESLESENILIKARNFLVFQGD
AN2963.1  	MAVYEDDAGEEQQWRRSITSQGVSEYRINNRVVTAQQYNEALEAENILIKARNFLVFQGD
          	******:**:**:*:*:**..* ******:****:* **::**:****************

MG06894.1 	VEAIANQSPQDLTRLIEQISGSLEHKPEYEKLKADLEQAAENQAFMLNRRRGINSEIRQY
NCU01323.1	VEAIASQSPQDLTRLIEQISGSLEYKADYEKLQAEVEQAAENQNFQLHRRRGINSEIKQY
FG01910.1 	VEAIASQSPQDLTRLIEQISGSLEYKAEYEKTQAESEQAAENQNFQLHRRRGINSEIKQY
AN2963.1  	VEAIASQSPKDLTRLIEQISGSLEYKAEYERLKAEAEESAEQQTLQLNRRRAINSEIKQY
          	*****.***:**************:*.:**: :*: *::**:* : *:***.*****:**

MG06894.1 	QEQKREAEAFQRKTEERDEAVVTQILWKLYHYQRIMDKSNEKINEHQENLKEFRRNVEAY
NCU01323.1	QEQKKEAENFQRKTEERDEAVITHILWKLYHFQKVMDESSAQIQEHQENLKEFRRNVEAF
FG01910.1 	REQKREADNFQNKTDERDAAIVTHSLWKLYHFQKAMDDSYAAIQDHQEDLKELRRNVESF
AN2963.1  	QEQKREAENYARKAEERDQAIITHILWKLFHFQRMIDDSSAEIQKYQDELKEYRRGVEKY
          	:***:**: : .*::*** *::*: ****:*:*: :*.*   *:.:*::*** **.** :

MG06894.1 	QRELEAAQREQAVVGRQVGKVERAIKEKEKAVEDEEGLLIPVETKIRETSQNVEARKRQL
NCU01323.1	ENKLDAARKEQATVGREVGKVERNIKAKEKDIEDKENSLVPIDEKIAQSTQDMGVLRKRI
FG01910.1 	EKRLDAARREQAAANRQVAMVDKDIKAKVRDIEDKENSLVPVEEKINESTEQVKALQVQV
AN2963.1  	ERNVEDAKKSHARVGRDVATAEKNIVKKEKEIEEATNALVPIDEKVDITKRKVERYSSRI
          	:..:: *::.:* ..*:*. .:: *  * : :*:  . *:*:: *:  :...:     ::

MG06894.1 	DSITKTRDEYAENVQKYKKELAKVEKAQQRFEAQWKEKLKNQGKELSDEDRKEYDDLRRQ
NCU01323.1	VEVKKDRDSQASSISKLQKDLATVEKAQQQFEKQWAETLKKQGKELSDEDRKEYTSLQAE
FG01910.1 	AKVTKEHDEQVEVVQQVQKSIESVEKAREIFENDYKEQMKKQGREVSDEDRREYSRLRTQ
AN2963.1  	AEIGKERDTQSASIKQLQKNLKVVEKAQAQWEADWQKALSKQGGQLSQADQQEYHKLKEE
          	 .: * :*     :.: :*.:  ****:  :* :: : :.:** ::*: *::**  *: :

MG06894.1 	ADIKSAENKSKLDVLTRQLKNDEATLAILARKTEQARTTVDKLNTEIEMLQRSLSDLQET
NCU01323.1	AMKKTADNRAKLANLTRQLKSDEVTVNSLKGKIDNFEAAIEKLQTEVQSIKDRKDASEDA
FG01910.1 	AMSRTGSNQAKLEQLDRQRKADEVTVNNLKGKVDSIAAAIDKIEAELASIDERRTSAQTI
AN2963.1  	VNKRSSAEQLKLDNLKRQRKTDAEAYNSLKSKFDSTQWQLQTLETDTKALSERKAAANET
          	.  ::. :: **  * ** * *  :   *  * :.    ::.::::   :.      :  

MG06894.1 	EGARTVEIEDKKKAFNNAQSERTRINQMRTQTEEKLREVLKRLEEADAGQRQTAKERNMK
NCU01323.1	VQQLRSDIAAKKKEYNKLQSERVRINQTRTAQEEKLREILRKLEDAESGRRQNEKETRLR
FG01910.1 	SKELSQEIDAKKKEFNQLQSERVRTNQKRTELEEKLEDVARKLREADDGRRQNDREARTK
AN2963.1  	VKTTSKEIEKKKKELNAISSERLRVSQMRTEYEEKLQVVLKKLLEADDGKKQTEREIRAK
          	      :*  ***  *  .*** * .* **  ****. : ::* :*: *::*. :* . :

MG06894.1 	ETISNLKRLYPGVKGRVGDLCRPKQKKFTDAVVIALGHDFDTIVVETNKVVDECLDYLKK
NCU01323.1	NMISDLRRIYPGVRGRVGDLCKPKQKKFDEAVITALGRDFDAVVVDTEKVGMDCVQYLKE
FG01910.1 	EMVTSLKRMFPGVRGRVGDLCKPKQKKFDEAVIVALGRDFDSVVVDSEKIGVECVQYLKE
AN2963.1  	ELISTLKRIFPGVKGRVSDLCKPKQKKYADAVSTVLGRHFDAIVVDNEKTAKECIQHLRD
          	: :: *:*::***:***.***:*****: :**  .**:.**::**:.:*   :*:::*:.

MG06894.1 	QRLPRMNFIPLDNIKASTPFAALKGKA-GVRLVIDTIDFDPSVERAMAYACGSSIVCDTF
NCU01323.1	QRFPPLTFIPLDNIKVNSSVSAVKGIS-GARLTIDTIDFDPSLERAISYACGGSVVCDNL
FG01910.1 	QRFPPMTFIPLDNIKVNAVNTAVKGFS-GARLTIDTIDFDSSVERAMSYACGSSVVCDSL
AN2963.1  	QRAGQATFIPLETIQVKALNSNLKGMHRAMRPAIETVDYDDSVARAITYACGNAIVCDDL
          	**    .****:.*:..:  : :**   . * .*:*:*:* *: **::****.::*** :

MG06894.1 	DIAKSICYDEKIAVKAVTLDGKLIHKGGLMTGGRLPDNKGNRRRFEDVDVQQLKDKASEY
NCU01323.1	HIAKDIVYGRKIQVKAVTLEGFVIHKAGTMTGGRLPNEKGGKRRFEEHDIQNLQRMAQSL
FG01910.1 	DIAKHICYEKKIPVKAVTLEGYIIHKAGLMTGGRGPESKN-KRRFEEADVQNLQRMATKL
AN2963.1  	ATAKYLCYERHVDAKAVTLDGTVIHKGGLMTGGRGPQQNS--KRWEDSEVESLFKLKDKI
          	  ** : * .:: .*****:* :***.* ***** *:.:.  :*:*: :::.*     . 

MG06894.1 	RSELEQLYQRDMQLRDRDGIREELAALERSVRGDRQELQALKKNLQSKQRELEHAEAQLA
NCU01323.1	KDEVAALAHSGRRTAKEDALLVEFTALEQRLKIQEGELAAFEKNLKSKQKELDHQERQLD
FG01910.1 	KDEIDRLPKADRRGSQEESLQIDLSGLERRLVAVKDEMAALNKNHASKKRELDNQRRQLN
AN2963.1  	MADLANLPKGHRRGTEEETLQGELVGLEQRLAYAREELKALERNLKDKYTELDFVKRQLE
          	  ::  * :   :  ..: :  :: .**: :   . *: *:::*  .*  **:  . ** 

MG06894.1 	DWESKAADKSGEVDTARKAVERFRKAIADVEDKIFADFCKRLGYNDIRAYEEQRGNLEAQ
NCU01323.1	DYEPKYEEKHGELERTRATVQKFEKAISDVEDKIFKDFCKRLGYENVRAYEAQQGTLEQE
FG01910.1 	ELEPKYQEQASQLESTTATCEEFRDAIARVDDEVFGDFCRRLGYSDIRAYRDSQGKLEQE
AN2963.1  	DLRPKYTEKQEVLDELDEAIATSQDAVSGVEDEIYRKFCKRLGYSNIREYEVQQGSLHEE
          	: ..*  ::   ::    :    ..*:: *:*::: .**:****.::* *. .:*.*. :

MG06894.1 	AASERSKFGDQQSKLSTTLKFEESRLANTEQRLQGVQREVERLEANIETYSQEEATIKDN
NCU01323.1	AAQKRQDFDIQKQRIQSNITWEMSQHTATSDRIASLERTLQRHERDLDTYRQEKASIEEE
FG01910.1 	VSEKRNEFDVQKQKLSTRLGWEQHRVAMSTGRIDTIQKQIRHLKKEIKTYTKAKDEIENA
AN2963.1  	AAQKKLEFTTQKSRIENQLSFEQQRLQATTDRIASLQAQHQRDANMIGELESEQERVRNK
          	.:.:: .*  *:.::.. : :*  :   :  *:  ::   .:    :    . :  :.: 

MG06894.1 	IETLNDELAQLRENLEGLKGEHKEKVVKAQEARSEVQTRTKDIDARQKAIDALETEVQKN
NCU01323.1	LAEDREALEELEQSLEELKVSHAEKTKKVQEAKQDLQRRSRDIEVRLKEISNLEATVQQS
FG01910.1 	LREEQEALEALRETLDENRSELAERNQKVSEAKVEVQKRSKDIDTHLRDINALETIVQKN
AN2963.1  	LDELNAELDILREKLEEQKEKYGQSAENLAHHRRELQKRSKEVESTLRFINGLETEIQRN
          	:   .  *  *.:.*:  : .  :   :  . : ::* *:::::   : *. **: :*:.

MG06894.1 	SAQKFSLLRRCKLERIQVPLKEGS--LDDLPDEDEILNQDPDAMDVDDDDD----GAMEA
NCU01323.1	SAQKLALLRRCKLEQIQIPLQQGS--LDDIPNEDMLLQKDQDAMDVDGEDEDQEAELLEA
FG01910.1 	SSSKSALLRRCRLEQIRIPLVNGT--LDNLPNEDELLRQDPDAMDIDEDDE----EMMDM
AN2963.1  	SSSRYALLRRCKLEDIDIPLTEESNPLDQLPIDELVQGADPDAMDVDEDTQ----ANGGF
          	*:.: :*****:** * :** : :..**::* :: :   * ****:* : :         

MG06894.1 	AMDDHGIAIDYDSLPEDLRD0SDEDGIEEQLERKISELNTELEKLNPNMRAVERLETVET
NCU01323.1	AMDDYGVEINYDNLDDALLQ~DPNDEVEEKLQEKISALTAEIEKLNPNMRAIERLESVKS
FG01910.1 	ALNDHGIAIDFDGLDDDLKA0SDDPSVEDGLSEKITSLTSELEKLNPNMRAMERLESVET
AN2963.1  	SVQDYGIEVDFDSLGDTLKE0DSDEKLEEELLEKVRTLNSELDKMAPNTRALERLESVEN
          	:::*:*: :::*.* : *   . :  :*: * .*:  *.:*::*: ** **:****:*:.

MG06894.1 	RLKQTDTEFQDAKVAYKNAHEAFEKIKNLRFEKFDKAFKHIQEQISSVYKELTRSDAYPL
NCU01323.1	RLESTEKDFEDSRAALKAARDAFNQVKDKRFELFNKAFTHIQEQITHVYKDLTRSDAYPL
FG01910.1 	RLRVTDQEYEDSKTAAQEAKEAFSEVKQKRYDLFNKAFSHIQEQISHVYKDLTRSEAYPL
AN2963.1  	KLRATEKDFEQARKRARKAKDDFEEVMRRRSELFNKAFSHISEQIGPIYRELTRSSNYPL
          	:*. *: ::::::   : *:: *.::   * : *:***.**.***  :*::****. ***

MG06894.1 	GGQA~YLDIEEDTDTPYLSGIKYHAMPPLKRFRDMEHLSGGEKTMAAMALLFAIHSFQPS
NCU01323.1	GGQA~YLDIEEDTDTPYLSGIKYHAMPPLKRFRDMEHLSGGEKTMAALALLFAIHSYQPS
FG01910.1 	GGQA~YLDIEEDTDMPYLSGIKYHAMPPLKRFRDMEHLSGGEKTMAALALLFAIHSYQPS
AN2963.1  	GGQA2YLDIED-SDEPYLDGIKYHAMPPLKRFRDMEHLSGGEKTMAALALLFAIHSYQPS
          	**** *****: :* ***.****************************:********:***

MG06894.1 	PFFVLDEVDAALDNANVDKIKKYIREHAGPGMQFIVISLKPGLFQDSESLVGVYRDQDVN
NCU01323.1	PFFVLDEVDAALDNANVEKIKKYIREHAGPGMQFIVISLKAGLFQDSESLVGVYRDQDVN
FG01910.1 	PFFVLDEVDAALDNANVDKIKKYIKDHRGPGMQFIVISLKAGLFQDSDSLVGVYRDQEVN
AN2963.1  	PFFVLDEVDAALDNTNVARIANYIYDHAAPGMQFIVISLKNGLFQNSEALVGIYRDQVEN
          	**************:** :* :** :* .*********** ****:*::***:****  *

MG06894.1 	SSRTMTLD0LR------PYQ---------
NCU01323.1	SSKTLTLD~VS----FSPPFLLLLTI---
FG01910.1 	SSRTLTLD0RIGRCMFGPSQHGGGMDGFM
AN2963.1  	SSKSLTLD0LR------KYT---------
          	**:::***
```
